# Implications of state policy context for the well‐being of immigrant families with young children

**DOI:** 10.1002/ajcp.12783

**Published:** 2025-01-15

**Authors:** Kevin Ferreira van Leer, Caitlin Lombardi, Rachel Chazan‐Cohen, Vanessa Esquivel, Prisila Isais, Anne Berset

**Affiliations:** ^1^ Human Development & Family Sciences University of Connecticut Storrs Connecticut USA; ^2^ California State University Sacramento California USA

**Keywords:** immigrant, low‐income, mixed methods, parenting, state policy, stress

## Abstract

There is notable variation in state‐level social policy exclusions for immigrant parents and their children. Little research has investigated how these exclusions impair the well‐being of immigrant families. This study examined how state‐level social policy exclusions for immigrants are associated with the well‐being of immigrant parents and development of their children. A mixed methods approach guided by the transformative framework was used with quantitative analyses among a subsample of low‐income immigrant parents from the Early Childhood Longitudinal Study—Birth Cohort (ECLS‐B; *N* = 1550) and qualitative focus groups with immigrant parents of young children from two states with differing social policy contexts: California (*n* = 18) and New Hampshire (*n* = 17). Results indicated that low‐income immigrant parents with young children experienced greater parenting‐related stressors in states with more restrictive policies toward immigrants. Quantitative findings revealed that children born in more exclusionary states had lower reading skills at age 4 and kindergarten. Findings from the qualitative focus groups identified a core category centered on *humanity being at the hands of the state*, with the following themes: (1) *salience of immigrant limitations*; (2) *state climate toward immigrants*; and (3) *social programs reduce stress, but access is variable and filled with barriers*. Policy and practice implications are discussed.

## Highlights

- Mixed methods used to examine the role of state policy exclusions on immigrant family well‐being.
- Immigrant parents in restrictive states had greater parental stress when children were 2 and 4.
- Qualitative results outline how sociopolitical contexts influence stress and social program access.

One in four US children lived with an immigrant parent in 2018 (Lou et al., 2019). Approximately half of these families have low‐incomes, in comparison to one‐third of non‐immigrant families (Lou et al., 2019). Nonetheless, there are lower rates of participation in means‐tested social programs for immigrant families with low‐incomes relative to non‐immigrant families with low‐incomes (Lacarte et al., 2024). Means‐tested social programs are those government assistance programs that determine eligibility based on household income and/or other indicators of household resources. Policies of exclusion, fear of stigma and deportation, and administrative burden have been theorized to explain these lower rates of utilization (Perreira & Pedroza, 2019). Lower access to social programs has implications for the well‐being of immigrant families, as these programs are shown to boost incomes, benefit families (National Academies of Sciences, Engineering, and Medicine, 2019), and promote equity and resilience (Vesely et al., 2017).

## POLICIES AFFECTING USE OF SOCIAL PROGRAMS BY IMMIGRANT FAMILIES

Changes to immigrants' eligibility for social service programs began with the passage of the Personal Responsibility and Work Opportunity Reconciliation Act of 1996 (PRWORA), which included a 5‐year bar for immigrants with legal permanent residency from accessing many large federally funded programs. This meant that immigrants with legal permanent residency were ineligible for programs, such as Medicaid and Supplemental Nutrition Assistance Program (SNAP), during their first 5 years. While exceptions have emerged over time for some programs and situations (e.g., pregnancy), the 5‐year bar largely remains. Lacarte et al. (2024) offers an overview of immigrants' federal eligibility for programs by immigration status and program, along with insights into specific state‐level policies. PRWORA introduced many restrictions; however, it also granted individual states the ability to determine immigrants' eligibility for programs. Since PRWORA's passage, states have had the flexibility to further restrict or expand coverage for immigrants, which they have done in varying ways.

Following the passage of PRWORA, there were dramatic declines in the use of social programs attributed to immigrant parents' reticence to access programs due to eligibility concerns or fear that program use would negatively impact their families' future opportunities (Yoshikawa & Kalil, 2011). It is important to note that while unauthorized immigrants have always been ineligible for these programs, many of their children are US citizens and are therefore eligible. Over two decades later, these fears have been heightened by changes to the “public charge rule,” which further disincentivized eligible immigrant parents from accessing public benefits (Perreira et al., 2018; Vesely et al., 2019). Public charge refers to the determination that an individual is primarily dependent on the government for subsistence, and thus would be inadmissible or ineligible for legal permanent resident status; specific government guidance is issued regarding public charge determinations, and which benefits may be considered. In 2019, the federal government widely expanded the list of government assistance programs which could be considered. The announcement of these changes was associated with decreased enrollment in Medicaid, SNAP, and WIC (Special Supplemental Nutrition Program for Women, Infants, and Children; Barofsky et al., 2020). While the expansion of which benefits were included was reversed in 2020, researchers have documented that immigrants forgo receiving social programs, even those excluded from public charge guidelines, due to their sensitivity to narratives of being viewed as a public burden, misinformation regarding eligibility, and concerns regarding future immigration status (Galletly et al., 2023). Scholars have outlined how these policies are part of larger systems that oppress immigrants in the United States (Buckingham et al., 2021).

### Access to social programs

Relative to non‐immigrant families, immigrant families with low‐income have lower rates of participation in means‐tested social programs, such as SNAP, WIC, and health insurance (Medicaid and the Children's Health Insurance Program). Furthermore, participation rates in these social programs varies widely across the United States (Lacarte et al., 2023; Lacarte, 2022; Perreira et al., 2012). Lower rates of utilization have been related to community and state anti‐immigration and exclusion, fear of stigma and deportation, and access barriers, such as language and program familiarity (Barajas‐Gonzalez et al., 2022; Perreira & Pedroza, 2019; Vesely et al., 2015). The multidimensional process of social exclusion, as defined by the United Nations Department of Economic & Social Affairs, (2016), involves being denied or having restricted access to full participation in society and the inability to utilize resources offered within that society. Social exclusion theory posits that lack of access to political, social, and health systems places additional strain on families beyond economic pressures, disadvantaging children's development (Yoshikawa et al., 2008). The lower rates of social program utilization, in addition to significant barriers described by immigrant families in accessing programs, reflects their shared experience of social exclusion. Given that social programs are state‐regulated, the process and experience of social exclusion could vary across states. Immigrants residing in states perceived as more generous toward immigrants, or providing greater access to social programs, could experience less social exclusion than immigrants living in states perceived as less generous (Perreira & Pedroza, 2019; Zimmerman & Tumlin, 1999).

### Social program support and immigrant family well‐being

A growing body of literature demonstrates that accessing and receiving social programs is associated with promoting the well‐being of immigrant adults. For example, among Latine male immigrant farmworkers, those who reported participating in SNAP or WIC also reported better health relative to those not receiving program benefits within the last 2 years (Rockler et al., 2022). These findings align with previous literature highlighting SNAP's positive impact on overall health (Gregory & Deb, 2015). Immigrant families who receive SNAP/WIC may have greater access to nutritious foods essential for health and well‐being. Conversely, fear of public charge deters some eligible immigrants from enrolling, using benefits for which they are enrolled, or re‐enrolling (Galletly et al., 2023). Less research has examined the impact of social program receipt on the development of children in immigrant families. One study found that parental use of social services moderated the relationship between parent legal vulnerability and children's reading and spelling skills (Brabeck et al., 2016), which highlights the potentially significant role of social services.

Given the evidence of the positive effects of social programs on immigrant health and well‐being, increasing access is essential. However, few studies have comprehensively explored how state‐level variation in program eligibility promote the well‐being of immigrant parents and development of their children. In this paper, we use a broad definition of well‐being, which includes stress and depressive symptoms for parents, as well as developmental domains for children, covering early cognitive skills (reading and math) and early behavioral skills (attention skills and externalizing problems).

### PRESENT STUDY

Access to social programs has been a longstanding concern for immigrant justice movements (Buckingham et al., 2021), providing an opportunity for research to inform ongoing efforts advocating for the inclusion of immigrants in social program eligibility. Thus, the present study investigates how state‐level social policy exclusions for immigrants are associated with the well‐being of immigrant parents and development of their children during early childhood. We draw on indicators of state‐level social policy exclusions for immigrants (Zimmerman & Tumlin, 1999). An explanatory mixed methods design was used, guided by the transformative framework. First, we conducted quantitative analyses using the Early Childhood Longitudinal Study—Birth Cohort (ECLS‐B; *N* = 1550). Second, qualitative focus groups were conducted with immigrant parents with young children in two states with differing policy contexts—California (CA; *n* = 18), a non‐exclusive state, and New Hampshire (NH; *n* = 17), an exclusive state—to better understand the first phase results. All households in the qualitative phase had received WIC, SNAP, Temporary Assistance for Needy Families (TANF), or Medicaid in the last year.

## METHODS

### Research design

**Figure 1:**
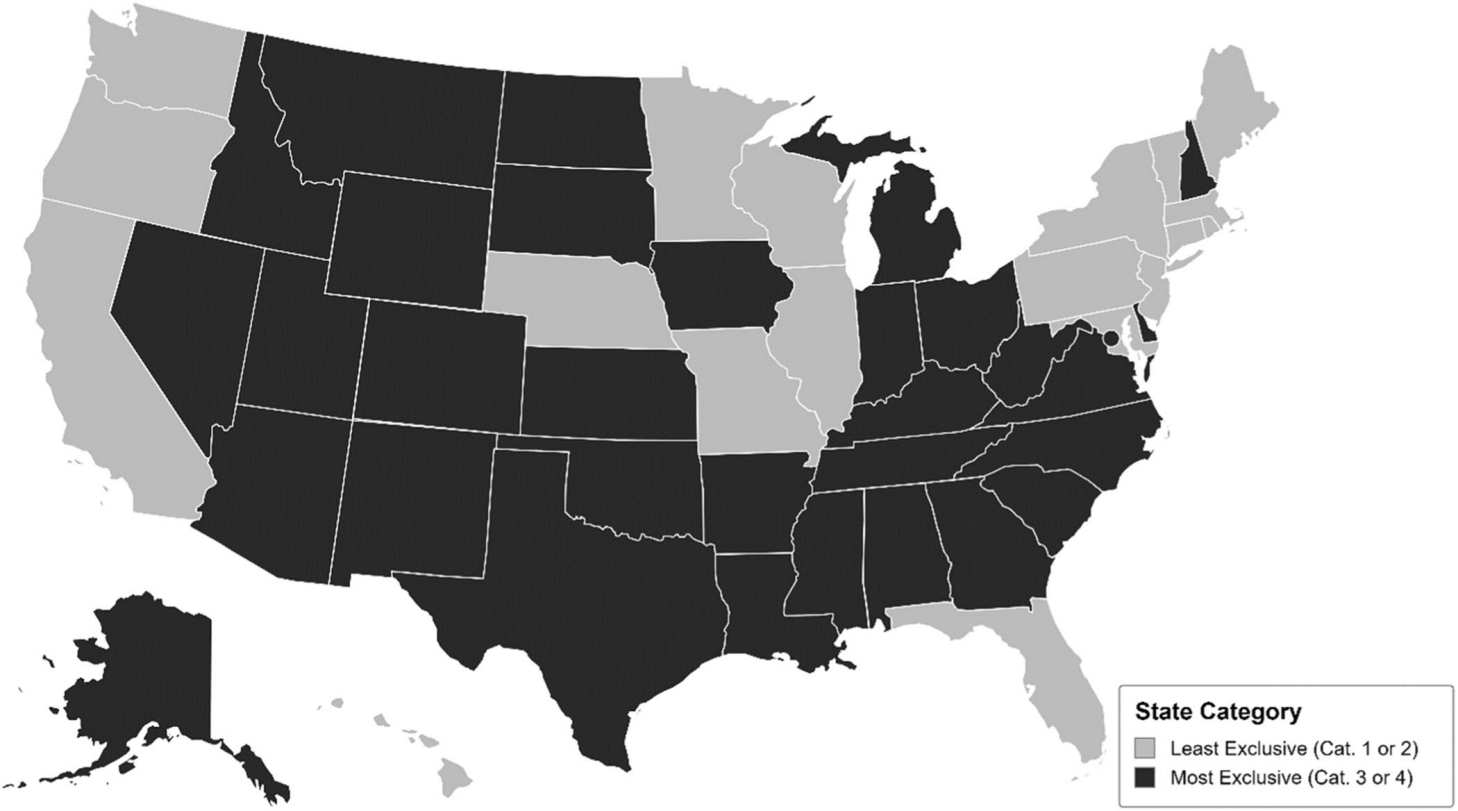
Map of exclusion category by state. States are categorized by their exclusionary policies toward immigrants using the rating system developed by Zimmerman and Tumlin (1999).

Undergirded by the transformative framework, which situates research in response to social injustices, this study explicitly sought to address inequities for immigrant families and support community processes for justice (Mertens, 2008, 2012). The transformative framework emphasizes centering the voices of community members throughout the research process. This was done in two ways. First, an advisory board made up of experts in policy, advocacy, and immigration‐related research, including individuals who identify as immigrant parents or are from immigrant families, provided insights into the study design, methods, and findings related to potential program and policy implications. Second, we partnered with two immigrant‐serving, community‐led organizations in CA and NH, where focus groups were conducted. Partnering with these organizations aligned with the transformative framework, enabling the qualitative phase of the project to consider community norms in data collection and analysis (Mertens, 2012). The two states were chosen based on (a) their historical and current differences in social policy exclusions for immigrants, (b) the presence of specific geographic communities with significant immigrant populations, and (c) established partnerships with community‐led organizations. The categories assigned to CA and NH for state restrictiveness toward immigrants in 1999 remained largely unchanged in 2023, using the point assignment system developed by Zimmerman and Tumlin (1999, see Figure 1).

Together, the advisory board and community‐based organizations informed the present study, which is part of a larger project investigating the role of social policies and community characteristics in promoting the well‐being of immigrant families throughout early childhood in the United States. The transformative framework asks researchers to ask critical questions of the research design and how it can support possibilities for change (Mertens, 2008). Thus, as part of the larger project, we aimed to share findings with partners and collaborate on community‐identified dissemination strategies to support their ongoing initiatives.

An explanatory mixed methods design was used. Phase 1 utilized quantitative methods to examine associations between living in a state with exclusive policies and parent well‐being and child development among low‐income immigrant families. Phase 2 used qualitative methods to gather the emic perspectives of immigrant parents with young children regarding their use of social programs in exclusive and non‐exclusive states, and its impact on their well‐being. Additionally, Phase 2 sought to explore parents' insights of Phase 1 findings. The findings from each phase were then integrated and examined in response to the research question.

### Phase 1: Quantitative study

#### Sample

We employed data from the Early Childhood Longitudinal Survey—Birth Cohort (ECLS‐B), a nationally representative sample of 10,700 children born in the United States in 2001. Births were sampled from 96 core primary sampling units, which were geographic regions consisting of counties or groups of counties. Children who died or were adopted before the age of 9 months and children born to mothers younger than 15 years were excluded from the sample. Data was collected at four time points based on child age: 9 months, 2 years, 4 years, and kindergarten entry. The response rate at 9 months was 74%; from the baseline 9‐month sample, the response rates for the 2‐year, 4‐year, and 5‐year waves of data were 93%, 91%, and 92%. Access to the ECLS‐B data was obtained by a restricted data license; more information on the study design and measures can be found in the user manual (Najarian et al., 2010).

This study utilized a subsample of 1,500 children from families that were low‐income (<200% of the Federal Poverty Line; FPL) and in which at least one parent reported being born outside of the United States. Missing data on individual measures and due to attrition was imputed in Stata 17 using multiple imputation. All descriptive statistics and analyses were weighted by jackknife replicate weights which adjust for sampling procedures, nonresponse, differential attrition, and properly adjust standard errors, making results generalizable to children born in the United States in 2001 to an immigrant parent. Sample descriptive statistics are shown in Table 1.

**Table 1:** Immigrant families with low‐incomes in the ECLS‐B, *N* = 1500.

	*M* (SD) or %
State policy exclusions toward immigrants	
1 or 2 (least)	66.51
3 or 4 (most)	33.49
State policy contexts, 2001	
Unemployment rate %	4.81
Earned Income Tax Credit indicator	28.96
Minimum wage in $	5.40 (0.35)
TANF/SNAP max for 3 in $	733 (127)
Child characteristics	
Male	50.29
Twin	10.78
Low birthweight	22.00
Race/ethnicity	
White	6.59
Black	6.34
Latine	59.41
Asian	24.58
Multi‐race	2.82
Child age wave 4/5 (months)	67.68 (4.14)
Family characteristics	
Maternal age at 9 months (years)	28.46 (6.27)
Parent education at 9 months (years)	12.57
Marital status at 9 months	66.37
Income at 9 months in $	21,579 (10,636)
Weekly hours employed at 9 months	17.32 (18.06)
# of children at 9 months	2.59 (1.34)
Age at immigration (years)	17.79 (8.77)
US citizen at 24 months	57.05
Household English proficiency	3.25 (1.31)
Parent well‐being	
Parenting stress age 24 months	2.88 (0.69)
Parenting stress age 4 years	2.85 (0.85)
Parenting stress kind.	2.86 (0.87)
Depressive symptoms age 4 years	5.91 (5.90)
Depressive symptoms kind.	5.16 (5.15)
Child development	
Reading skills age 4 years	21.14 (9.97)
Math skills age 4 years	26.61 (10.41)
Reading skills kind.	38.51 (15.48)
Math skills kind.	40.29 (11.15)
Externalizing problems age 4	2.39 (0.69)
Attention skills age 4 years	3.77 (0.69)
Externalizing problems kind	2.25 (0.71)
Attention skills kind	3.87 (0.63)

*Note*: All values are weighted with sample weights.

*Source*: US Department of Education, National Center for Education Statistics, Early Childhood Longitudinal Study, Birth Cohort (ECLS‐B), 9‐month, 2‐year, 4‐year, and kindergarten data collection.

The ECSL‐B provides significant strengths in answering the research question; the data are nationally representative, were collected in multiple languages, and there is a sizable subsample of children from low‐income families with one or more immigrant parents. Furthermore, the data collection occurred in the wake of shifts to policy eligibility for immigrant families that occurred with the passage of PRWORA in 1996. At the same time, there are notable weaknesses to this sample given that it is now over two decades old.

#### Measures

##### State policies exclusions toward immigrants

To measure the *exclusionary state policies toward immigrants*, we utilized a rating system developed by Zimmerman and Tumlin (1999). To understand the impact of the passage of PWORA in 1996 for immigrants, Zimmerman and Tumlin (1999) examined state policies across state food, cash assistance, and health insurance policies and programs, as well as efforts to promote state naturalization initiatives. Zimmerman and Tumlin (1999) grouped states into four categories (*4 = most generous to 1 = least generous*) that represented immigrant access to state safety nets. This variable was reverse coded and combined into indicators to indicate most exclusionary (*3 and 4*) to no exclusions (*1 and 2*). See Figure 1 for state indicators. Families were coded with the exclusion variable that corresponds to their state of residence at wave 1 when the child was 9 months old.

##### Parent well‐being

Two measures assessed parents' report of their own well‐being, which were most often reported by biological mothers. First, parents reported on their *depressive symptoms* using 12 items drawn from the Center for Epidemiological Studies‐Depression scale (Radloff, 1977). Items were scored on a 4‐point scale (*1 = never to 4 = most of the time*; *α* = .88), with higher scores indicating more depressive symptoms. Second, parents reported on their *parenting stress*, with 6 items drawn from the Parenting Stress Index (Abidin, 1983) that were responded to on 4‐point scales (*1 = never to 4 = most of the time*; *α* = .62). The measure asked parents to report on the difficulties they experience as a parent, with higher scores indicating more stress.

##### Children's development

Children's *reading and math skills* at ages 4 years and kindergarten were measured using direct assessments comprised of items drawn from well‐validated standardized instruments including the Peabody Picture Vocabulary Test Third Edition (PPVT‐III) (Dunn & Dunn, 1997), the PreLAS 2000 (Duncan & DeAvila, 1998), the Preschool Comprehensive Test of Phonological & Print Processing (Lonigan et al., 2002), and the Test of Early Mathematics Ability (3rd ed.; Ginsburg & Baroody, 2003). The reading assessment (*α* = .92) consisted of 74 items that measured early reading and language skills. The math assessment (*α* = .92) consisted of 58 items focused on number sense, operations, and probability.

At ages 4 years and kindergarten, children's *externalizing problems and attention skills* were measured with parent‐reported items from the Preschool‐Kindergarten Behavior Scales (Merrell, 2003) and Social Skills Rating System (Gresham & Elliott, 1990). A composite of externalizing problems assessed children's impulsive, disruptive, and aggressive behaviors (seven items; *α*_3_ = .69, *α*_4–5_ = .74). A composite of attention skills assessed children's attention, independence, task completion, and eagerness to learn (four items, *α*_3_ = .58; eight items, *α*_4–5_ = .74).

##### State policy context in 2001

To adjust for other characteristics of states that may be related to the policy context and also parent well‐being and child development, we included state‐level data from 2001 on the state unemployment rate, whether the state had adopted an Earned Income Tax Credit (EITC), the minimum wage, and the maximum monthly combined TANF and SNAP for a family of three.

##### Child and family characteristics

A carefully selected collection of child and family characteristics were included in the statistical models to rule out potential confounds. These characteristics were chosen based upon previous similar research examining immigrant family well‐being and child development (e.g., Brabeck et al., 2016; Vesely et al., 2015). Child characteristics included age in months at kindergarten assessment, sex (an indicator for male), race/ethnicity (non‐Hispanic White, non‐Hispanic Black, Native Hispanic, Immigrant Hispanic, Asian, American Indian, and multiracial), an indicator for children who entered kindergarten, an indicator for low birthweight status, and an indicator for being from a multiple birth. Family characteristics, all measured at 9 months, were comprised of maternal age, income, highest parental years of education, marital status, average weekly hours employed by parents, and number of children in the household. At 24 months, parents reported if they were a US citizen and the age in years that they immigrated to the United States. Finally, we included a continuous measure of household English proficiency, which was comprised of an averaged composite of responses to how well parents could read, write, speak, and understand English (1 =* not well at all* to 4 =* very well*) at the 9‐month interview.

#### Phase 1 analytical plan

To examine state‐level social policy exclusions for immigrants as a predictor of parent well‐being and child development, ordinary least squares (OLS) regression models were estimated. The state policy exclusion indicator was used to predict each parent and child outcome, adjusting for other state‐level policy indicators and child and family characteristics. Separate models were run for each outcome measure.

### Phase 2: Qualitative study

#### Data collection

Following the typology used in Phase 1, data were collected from two states: (1) California, representing non‐exclusive policies, and (2) New Hampshire, representing exclusive policies. Recruitment was facilitated by the partner community‐led organization in each state through in‐person conversations and sharing flyers. To ensure participants in this study could describe their experience with program utilization or lack thereof, we recruited individuals who had utilized social programs within the past year. The partner organizations hosted in‐person and virtual focus groups and provided feedback on the project.

Focus groups were used to gain emic perspectives of social program use, state policy exclusions, and family well‐being. Participants were asked to share what they found most important to their families' well‐being and their children's development. They were also asked to share their experience in their communities and how they utilize local resources and social programs. Participants were then asked to assist in interpreting differences between parent and child outcomes in relation to state exclusivity found in Phase 1. For example, the protocol included the question, “drawing on your experience, why do you think living in a state that might be more/less exclusive would be related to lower/higher stress parents?” Participants in NH were asked about being in a “more exclusive” state while participants in CA were asked about being in a “less exclusive” state. Visual aids were used to help participants understand state exclusivity and conceptualize ideas like parental stress.^1^ Previous researchers comparing interview and focus group methods have found that, when examined on a per‐person basis, individuals disclosed sensitive information more often in focus group settings (Guest et al., 2017). Thus, while discussing social program use or family stress may be a particularly sensitive topic, we felt confident that this data collection method would allow for some disclosure.

The focus groups were held in either English (*n* = 1) or Spanish (*n* = 8), determined in advance based on participants' comfort with either language. In CA, three focus groups were in‐person at a community partner location and three were virtual. In NH, all focus groups were virtual, and participants joined from their own locations. Audio was recorded and transcribed by members of the research team fluent in English, or English and Spanish, as appropriate. The researchers transcribing and analyzing focus groups in Spanish were also from Latine cultural groups and have previously conducted research with Latine populations. On average, focus groups lasted 1 h and 46 min.

#### Participants

Thirty‐five immigrant mothers with children under 5 years old participated in focus groups during the spring and summer of 2023. In CA, 18 mothers participated in 6 focus groups, and, in NH, 17 mothers participated across 3 focus groups. Participants were from Mexico, Dominican Republic, Colombia, El Salvador, Russia, and Ukraine. Of the 35 immigrant mothers, 29 participants shared their demographic information (*M*_*age*_ = 35, range = 26–44; see Table 2). Given participants were all immigrant mothers, we use the terms mothers, interviewees, and participants interchangeably when describing the findings.

**Table 2:** Phase 2: Participant demographic information.

	California mothers (*n* = 16)^a^	New Hampshire mothers (*n* = 13)^a^	Total mothers (*n* = 29)
Use of Government programs (within last year)			
WIC	14	6	20
CalFresh or SNAP	4	3	7
TANF, CalWorks, or FANF	0^b^	0	0^b^
MediCal or Medicaid	14	6	20
Language(s) spoken			
Spanish	14	12	26
English	6	0	6
Russian	1	0	1
Ukrainian	1	0	1
Maternal employment			
Full time employment	0	3	3
Part time employment	1	1	2
Not employed	15	8	23
Maternal country of origin			
California^c^	1	0	1
Colombia	0	2	2
Dominican Republic	0	7	7
El Salvador	1	0	1
Mexico	12	3	15
Russia	1	0	1
Ukraine	1	0	1
Number of years in United States			
1–5 years	3	3	6
6–10 years	2	5	7
10–15 years	2	2	4
15–20 years	3	1	4
More than 20 years	6	1	7
Child characteristics			
Live with partner/spouse (Yes)	16	5	21
Avg. number of people in house	5	4	5 (2–9)
Avg. number of children	2.81	2.54	2.69 (1–7)
Avg. child age (years)	8 (1–24 yo)	8 (4 mo–20 yo)	8 (4 mo–24 yo)
Number of children ≤ 5yo	20	18	38

^a^ Two participants decided not to share their demographic information from the Sacramento focus groups, and four from New Hampshire.

^b^ Two participants reported during focus groups receiving TANF more than 1 year ago.

^c^ Partner was born outside of the United States.

#### Data analysis

Two of the authors conducted data analysis using an inductive, modified constructivist grounded theory approach (Charmaz, 2014). Using a subset of transcripts from four focus groups, analysis began with line‐by‐line coding to apply a short descriptive code to each line of the transcript (Charmaz, 2014). The two researchers overlapped in coding two of these four transcripts and met to discuss their codes to examine similarities and differences in coding to ensure correspondence across coders. Any differences in coding were discussed and final codes were agreed upon. Line‐by‐line coding was then conducted for responses concerning personal and family well‐being, as well as participants' experiences related to associations found between parenting stress and state exclusivity in Phase 1 for all the remaining focus groups. Gerund codes were primarily utilized in line‐by‐line coding to capture the active nature of state exclusivity and its impact on families. Line‐by‐line codes were then grouped into axial codes or larger categories. Throughout the analysis process, the researchers engaged in constant comparison, an iterative process of comparing emergent themes to line‐by‐line codes and transcripts to ensure the themes were reflected across focus groups and accurately reflected the experiences of participants. Through this iterative process, supported by memo writing, a final set of themes were identified. Particular attention was paid to how themes emerged across or within focus groups by state, aligning with the research question. Additionally, the researchers conducting the analysis shared their emerging themes with the larger research team to conduct peer debriefing to further enhance trustworthiness (Lincoln & Guba, 1985). The final themes are presented below.

## RESULTS

### Phase 1: Quantitative findings

To examine state‐level social policy exclusions for immigrants as a predictor of parent well‐being and child development, a series of OLS regression models were estimated (Table 3). The state policy exclusion indicator was used to predict each parent and child outcome, adjusting for other state‐level policy indicators and child and family characteristics. Results in the first section of Table 3 indicate that parents living in a state with exclusive policies toward immigrants had higher parenting stress when their children were ages 24 months and 4 years. These differences were moderate in size, with both representing 0.26 standard deviations units (SDs). No significant associations emerged in relation to parenting stress when children were in kindergarten or parental depressive symptoms at ages 4 years or kindergarten.

**Table 3:** Associations between living in a state with exclusive policies toward immigrants and parent well‐being and child development for immigrant families with low‐income, *N* = 1,500.

	Living in a state with exclusive policies toward immigrants
Parent well‐being	
Parenting stress age 24 months	0.18 (0.08)*
Parenting stress age 4	0.22 (0.10)*
Parenting stress kind	0.08 (0.11)
Depressive symptoms age 4	0.38 (0.64)
Depressive symptoms kind	−0.18 (0.80)
Child development	
Reading skills age 4 years	−2.90 (1.15)*
Reading skills kind	−3.74 (1.65)*
Math skills age 4 years	−2.28 (1.38)
Math skills kind	−1.42 (1.23)
Externalizing problems age 4 years	−0.13 (0.08)
Externalizing problems kind	0.07 (0.09)
Attention skills age 4 years	−0.04 (0.08)
Attention skills kind	0.03 (0.07)

*Note*: All values are weighted with sample weights. All models control for child sex, twin, low birthweight, race/ethnicity, maternal age, highest parental education, marital status, income, parent employment hours, number of children, age at immigration, citizenship, and English proficiency, all measured at age 9 or 24 months. They also control for the state unemployment rate, an indicator for if the state had the Earned Income Tax Credit, minimum wage, and the max SNAP and TANF benefits for a family of 3, all measured in 2001.

*p < .05.

Turning to associations between living in a state with exclusive policies toward immigrants and children's development, living in an exclusive policy state was associated with lower reading skills at ages 4 years (SD = 0.29) and kindergarten (SD = 0.24). Living in a state with exclusive policies toward immigrants was not associated with children's math skills, externalizing problems, or attention skills at ages 4 and kindergarten.

### Phase 2: Qualitative findings

Focus groups provided valuable insights into the differing state climates in exclusive and non‐exclusive states. Participants identified several factors affecting their well‐being, including stress brought on by financial hardship, the salience of their immigration status, and state climate. A core category, *humanity being at the hands of the state*, emerged across focus groups, illustrating immigrant mothers' experiences with accessing and participating in social programs across both exclusive and non‐exclusive states. Participants frequently described how they felt “treated” by their respective state of residence. Participants in CA (non‐exclusive) expressed feeling supported by the state, which allowed them to feel recognized as human. In contrast, participants in NH (exclusive) perceived little to no support from the state and described feeling objectified rather than humanized, reflecting the salient challenges faced by immigrant families. These challenges were stark reminders for NH participants of their immigrant identity and the lack of support that comes with that, despite their humanity. The concept of *humanity being at the hands of the state* illustrates the perceived ability states have in reducing immigrant families to simply their immigration status, rather than caring for their well‐being and humanity. Three themes were identified that encompassed the core category of *humanity at the hands of the state*: (1) *salience of immigrant limitations*, (2) *state climate toward immigrants*, and (3) s*ocial programs reduce stress, but access is variable and filled with barriers*.

With respect to the core category of their *humanity being at the hands of the state*, mothers discussed the ways in which they were continually othered as immigrants and spoke to the ways that their humanity was recognized, or not, through interactions with individuals, social service agencies, and the larger state context. One interviewee from CA said, “I am in a country with many opportunities that my country doesn't have, but I feel useless.” Differences emerged between CA and NH focus groups in how participants experienced state restrictiveness and described feeling dehumanized. In NH, participants described a continuous sense of being othered—by individuals, employers, social service agencies, and state policies—while CA focus groups centered on discussions about inclusion and feeling valued by the state. One mother in NH shared, “I cried alone. Sometimes I wanted to run, and you know, sometimes being in your house locked in.” The mother goes on to share how the constraints of the state did not allow her to work, to be human. Another mother remarked on the NH state motto, sharing, “you know what the state says, ‘live free or die,’ you are screwed. So, I said, okay, leave it to me to do all I can on my own.” Overall, participants described their desire to provide for their families but felt burdened by the financial hardship of meeting basic needs and enrichment for their children. Indeed, participants highlighted how daily interactions with individuals and institutions simultaneously facilitated their desires and burdens. Three themes stem from this core category and are described below.

The *salience of immigrant limitations* emerged as a theme from participants awareness of their immigration status and the limitations that follow. For example, one mother from CA shared, “We are more affected than them [citizens]… For us, if we do not pay rent, they will throw us out, we know we have to pay on a certain day… there is pressure from that.” Limitations included perceiving a lack of available or accessible support services for different immigrant groups, as well as feelings of having to fend for oneself and abandoning dreams and aspirations. One parent from NH recounted:

Migrants come with dreams that their children become athletes, artists… they have the dream of giving everything to their children and they have their full intention of supporting their children. But it is very difficult, and it ends along the way, like a failure, no not a failure, more like they forget that initial dream for their children because it becomes unattainable.

Questions regarding public charge often arose across focus groups where participants worried about the potential consequence of utilizing social programs for themselves or their children. Participants expressed uncertainty about policies related to public charge, and frequently discussed and disagreed on program eligibility or how program use might impact their immigration status. Overall, this demonstrates participants constant consideration and concern about which resources they could safely access.

These limitations were exacerbated by the state context, as participants spoke to the *state climate toward immigrants*. This theme encapsulates how participants perceived the level of openness toward immigrants within state policies as well as interpersonal interactions with organizations, agencies, or individuals in the state. Regarding state policy, CA participants highlighted the ways that they felt the state was responsive to their needs and often sought to include immigrant communities in relief or social service programs. For example, one focus group in CA, which was conducted 1 month after severe storms and flooding, noted that the state included unauthorized and other immigrant populations in state relief programs to support those affected by the storms. Participants in CA said that living in states with a different approach and limited resources might create more stress. One CA participant remarked, “Thanks be to God that we are in a state where they do like us.” In multiple focus groups, recent examples of state‐level anti‐immigrant policy changes arose, such as those recently passed in Florida, noting that these states might provide less support and create a more hostile environment for immigrant families. Moreover, participants described incidents of racism they encountered within the state. Again, there were notable differences between focus groups, with greater discussions of perceived interpersonal racism within the NH focus groups. As one participant from NH described, “But really, as a Latina, such as in my case, many, many times they treat you as though they look down on you.”

Lastly, participants across states described how *social programs reduce stress, however access is variable and filled with barriers*. Many participants spoke about the ways in which WIC, SNAP and Medicaid programs supported their access to basic needs, provided them opportunities to connect with other mothers, or shared information that supported their parenting. Not all participants were enrolled in every program, and there was a range of attitudes toward using different social programs across participants. For example, one participant shared that she chose not to enroll in food stamps, despite being eligible, but did enroll in WIC. Most participants were unfamiliar with TANF and questioned their eligibility. When discussing the programs families had utilized, participants overwhelmingly described how these programs provided relief from stress by alleviating financial hardship. In one instance, two participants from CA described this stress, with one stating, “sometimes you do not know if you should go buy food or pay a bill,” while another interjected, “and that is pure stress.” The first mother went on to say, “That is what I am saying, if you aren't going to become sick from an illness, you are going to get sick from the stress.” Indeed, some participants noted they had more time with their children and families because they had fewer difficult financial decisions or worries about financial stress. One participant from CA said:

And with food stamps, you grab [all your] kids, grab your list, and let's go. You only search if you're looking for lower prices, but you don't have to look to cut anything off the list and sometimes you can even have the luxury of ‘oh, grab some cookies, grab a juice,’ so, grocery shopping becomes family time.

This demonstrates how receiving food stamps alleviated financial stress and allowed for an enjoyable, rather than stressful, family outing. Yet participants identified several barriers to accessing or utilizing programs, including misinformation, language barriers (even when able to communicate well in English), and perceived discriminatory attitudes. Discussing her experience with a White caseworker, one participant from NH shared “they had to give me an interpreter, and I saw she [the case worker] was annoyed because I had been many times…The interpreter just looked at me, as if to say sorry, I am only translating.”

Together, these themes inform our understanding of participants' perceptions of state variation in social policy exclusions and the collective impact on immigrant family's well‐being. The *salience of immigrant limitations* theme outlines how participants described being continuously aware of their immigration status and how it limited their participation in society, including social programs, and aspirations. Additionally, parents described their perceptions of the state they resided in as welcoming, or not, to immigrants, which impacted interactions within the state. A handful of participants had moved across states and commented on their perceived differences in how states handled enrollment into programs, illustrating how the *salience of immigrant limitations* and *state climate toward immigrants* themes build upon each other. When comparing her previous experience in a less exclusive state to her current experience in NH, one participant stated “Yes, in the [less exclusive state] I received [food stamps] well and they gave me all the help.…. here [in NH] the process is stricter…. here the processes are, like, harder, more exhausting.” Families also discussed how enrollment paperwork was more tedious in exclusive states. Thus, some families recognized the discrepancies in program enrollment criteria and processes across states. Participants described social programs as supportive, as emphasized in the theme *social programs reduce stress, however access is variable and filled with barriers*. One CA mother encapsulates the impact of the programs she has enrolled in:

It is also that if you are not doing well yourself, very stressed, you don't have what you need that day to buy food, and you stress, you stress, and you are in a bad mood… if you receive that support… it is like economic relief… These programs, I repeat, are not only about here it [the resource] is, but it is look, here you can do this with it [what you receive]. In the case of WIC, they have given me menus, like look, you can change the way you make them [children] food.

In summary, these themes illustrate how participants perceptions of their state climate toward immigrants and the limitations of their immigration status constrained their use of social programs, which were discussed as reducing stress and supporting families.

### Side‐by‐side analysis

Findings across the quantitative data (Phase 1) and qualitative focus group data (Phase 2) were examined for discrepancies and similarities as well as how state variation in policy exclusions for immigrants are associated with immigrant family well‐being and child development (Table 4). Looking across phases, we found multiple areas of agreement. First, Phase 1 results reveal that families living in states with higher social policy exclusions had greater parenting stress when their children were 24 months and 4 years old. This association is represented within the following qualitative themes: *social programs reduce stress, however, access is variable and filled with barriers*, and *state climate toward immigrants*. These themes highlight the psychological hardship families encounter in fulfilling basic needs and demonstrate the benefits of accessible social service programs in reducing stress related to feeling forced to choose between essential needs. Indeed, state‐level variations in policies toward immigrants were noticeable to parents, which may have further exacerbated stress and perceived alienation. In light of these findings, uncertainties remain regarding factors influencing program utilization among families living in states with greater policy exclusions.

**Table 4:** Side‐by‐side analysis of quantitative and qualitative results.

Phase 1: Quantitative results	Phase 2: Qualitative themes	Congruent/discrepant
Families in states with higher social policy exclusions had higher parenting stress at ages 24 months and 4 years	Social programs reduce stress, but access is variable and filled with barriers State climate toward immigrants	**Similarities:** Stress evident across both phases. Program utilization reduces financial and psychological stress.
		**Divergences/Nuances:** State exclusivity may point to access to and acceptance of use of programs but does not necessarily lead to program utilization.
Children in states with higher social policy exclusions had lower reading skills at ages 4 years and kindergarten entry	Social programs reduce stress but access is variable and filled with barriers	**Similarities:** Partial support to describe mechanisms to understand quantitative associations in qualitative themes.
		**Divergences/Nuances:** Quantitative results revealed null associations for some measured constructs, differing from what might be expected from the ways in which families discussed the role of programs and state exclusions on stress and how their stress impacts their parenting.
Not applicable	Salience of immigrant limitations State climate toward immigrants	**Similarities:** Not applicable
		**Divergences/Nuances:** Focus group data suggest racial and xenophobic state climate as well as larger considerations regarding immigrations status may impact well‐being beyond state policy exclusions.

Second, there are similarities between Phases in how state policy exclusions for immigrant parents are related to child development. In Phase 1, we found that children living in states with greater social policy exclusions had lower reading scores at age 4. This finding is partially supported by parents' descriptions within the theme *social programs reduce stress, however access is variable and filled with barriers*. With support provided by social programs, parents described experiencing reduced stress and an enhanced ability to dedicate more time and resources to their children. However, mothers in focus groups did not explicitly mention utilizing this extra time to support reading or other educational endeavors. Although families may engage in these activities with their children, Phase 2 lacks a clear explanation for the association found in Phase 1 between state policy exclusions and reading scores at ages 4 and kindergarten, as well as the null results for other child outcomes. Phase 1 findings may not fully reflect families' experiences and coping mechanisms of pervasive stress, based on their accounts of social programs and state exclusions in Phase 2. Lastly, the core category identified in Phase 2—*humanity at the hands of the state*—did not have corresponding findings in Phase 1. This suggests the emergence of a broader phenomenon not captured by Phase 1 measures, which may reflect the multifaceted impact of state climate on well‐being.

## DISCUSSION

Findings illustrate that immigrant parents with young children and low‐incomes face parenting‐related stressors associated with state restrictiveness toward immigrants. Specifically, quantitative results in Phase 1 utilizing a national data set revealed that immigrant parents who lived in exclusive states in 2001 reported greater parental stress when their children were 2 and 4 years old. Paralleling these results two decades later, mothers in focus groups in Phase 2 highlighted the stress brought on by their state's climate toward immigrants, alongside challenges like meeting basic needs and persistent barriers to accessing social programs. These findings build upon the literature showcasing how immigrant parents often face stress via different societal contexts (Yoshikawa & Kalil, 2011). While parenting stress was experienced across focus group participants, it was particularly salient for those in NH with greater state‐policy restrictions. The difference in experiences was evident as immigrant mothers from CA expressed feeling cared for by their state, while mothers from NH frequently felt limited and unsupported.

Findings from Phase 2 elucidate how state and national sociopolitical contexts influence immigrant perceptions of access to and utilization of social programs, as well as the stress experienced by immigrant families. First, Phase 2 findings highlight the fear immigrant families experience when accessing social services and the future impact on immigration status. This aligns with emerging scholarship on how changes to the public‐charge rule have created reticence in accessing public programs among immigrant communities (Perreira et al., 2018; Vesely et al., 2019). Second, focus group data revealed a heightened negative racial and immigration climate in the exclusive state, which contributed to barriers in accessing programs and increased stress. These findings dovetail with research on the impact of immigration‐related threat on stress and well‐being for immigrant households (Roche et al., 2018) and children (Barajas‐Gonzalez et al., 2022). The salient challenges and fears of future repercussions described by immigrant parents in this study, and well‐noted in the literature, reflect the multi‐dimensional processes of social exclusion theory. Following the first Trump administration, immigrant families continue to face pervasive systemic barriers that hinder equal access to available resources and full participation in society (Bernstein et al., 2022). Ultimately, findings suggest that racist and xenophobic state climates, coupled with varying societal perceptions of immigrants across states, may impact immigrant family well‐being and children's well‐being beyond state policy exclusions.

### Study strengths and limitations

We utilized an explanatory mixed methods approach to capitalize on the strength of a longitudinal, representative sample of immigrant families with low‐income, coupled with perspectives from focus groups composed of contemporary immigrant families. By integrating the findings, we gained a holistic understanding of the ways in which state‐level policy exclusions were associated with the healthy functioning of children and parents in low‐income immigrant families in the United States. Aligning with the aims of the transformative framework, this research design allowed us to gain unique insight into a critical social issue of complex processes of oppression that immigrant families face.

To contextualize these findings, it is important to describe the limitations of this study. Although the quantitative findings come from the ECLS‐B, a nationally representative survey, these data are now over two decades old. During this time, there have been dramatic changes to social policies and programs and movements impacting immigrant communities (Barajas‐Gonzalez et al., 2022; Lacarte et al., 2024; Perreira & Pedroza, 2019; Perreira et al., 2012; Vesely et al., 2015). While developmental mechanisms remain similar, the policy, immigration, community, educational, and home environment contexts influencing children's development and parent well‐being are different across the two phases of the study.

We designed our explanatory mixed methods approach to address changes in the sociopolitical context. Indeed, our qualitative data collection examined the current immigration and policy context and explored how changes in the recent historical context might impact the interpretation and meaning of quantitative findings. However, we note caution regarding the transferability of the qualitative findings to all state contexts, as focus group data collection was limited to two states, CA and NH, which may be unique among non‐exclusive and exclusive states. Thus, further exploration of themes among immigrant parents with young children and low‐incomes in other state policy contexts would enhance our understanding.

In this study, we integrated several aspects of Phase 1 into the design and analysis of Phase 2, including asking participants about their interpretations of Phase 1 findings. However, there were several differences in the measures and sample characteristics preventing full integration. We were limited in the scope of measures in the ECLS‐B. For example, we were unable to examine parental perceptions of the state climate toward immigrants. More work needs to be done to examine the well‐being of low‐income immigrant families in the current political context. This study does not encompass the experiences of all immigrant families in the United States and findings are correlational, so interpretation and generalization may be limited.

### Policy implications and conclusions

In the context of these limitations, results from this mixed‐methods study yield several implications for policy and practice to support the well‐being of immigrant parents with young children and low‐income. First, it is important for state policymakers to understand how state‐level policies impact immigrant families' daily lives. When proposing policy changes, policymakers should consider how they could limit immigrant families, across different immigration statuses, and impose added stress. Understanding the impact of state‐level policies is even more crucial for states that are more exclusive toward immigrants and thereby the state climate may already create stressors for immigrant family well‐being. Given project findings, policymakers in more exclusive states should reassess existing social program policies and consider expanding eligibility for immigrant families. In this study, access to social programs was found to be associated with positive developmental outcomes, highlighting the importance of ensuring eligible families receive the benefits of social programs. Study findings illustrate the potential consequences of structural inequities, namely state exclusivity for immigrants to social programs.

## CONFLICT OF INTEREST STATEMENT

The authors declare no conflicts of interest.

## Data Availability

The participants of this study did not give consent for their data to be shared publicly, so due to the sensitive nature of the research, supporting data is not available.

## Footnotes

1 Available by request.
